# CCDC88C, an O-GalNAc glycosylation substrate of GALNT6, drives breast cancer metastasis by promoting c-JUN-mediated *CEMIP* transcription

**DOI:** 10.1186/s12935-024-03413-2

**Published:** 2024-07-06

**Authors:** Boya Deng, Siyang Zhang, Yingying Zhou, Ting Sun, Ying Zhu, Jing Fei, Ailin Li, Yuan Miao

**Affiliations:** 1 Department of Gynecology, The Second Affiliated Hospital of Zhejiang University, Hangzhou, Zhejiang China; 2 Science Experimental Center of China Medical University, Shenyang, Liaoning China; 3 Department of Obstetrics and Gynecology, Shengjing Hospital of China Medical University, Shenyang, Liaoning China; 4 Department of Radiotherapy, Cancer Hospital of China Medical University, Liaoning Cancer Hospital & Institute, Cancer Hospital of Dalian University of Technology, Shenyang, Liaoning China; 5 Department of Pathology, The College of Basic Medicine Science and the First Hospital of China Medical University, Shenyang, Liaoning China

**Keywords:** Breast cancer, CCDC88C, GALNT6, JUN, CEMIP, Metastasis

## Abstract

Coiled-coil domain containing 88C (CCDC88C) is a component of non-canonical Wnt signaling, and its dysregulation causes colorectal cancer metastasis. Dysregulated expression of CCDC88C was observed in lymph node metastatic tumor tissues of breast cancer. However, the role of CCDC88C in breast cancer metastasis remains unclear. To address this, the stable BT549 and SKBR3 cell lines with CCDC88C overexpression or knockdown were developed. Loss/gain-of-function experiments suggested that CCDC88C drove breast cancer cell motility in vitro and lung and liver metastasis in vivo. We found that CCDC88C led to c-JUN-induced transcription activation. Overlapping genes were identified from the genes modulated by CCDC88C and c-JUN. *CEMIP*, one of these overlapping genes, has been confirmed to confer breast cancer metastasis. We found that CCDC88C regulated *CEMIP* mRNA levels via c-JUN and it exerted pro-metastatic capabilities in a CEMIP-dependent manner. Moreover, we identified the CCDC88C as a substrate of polypeptide N-acetylgalactosaminyltransferase 6 (GALNT6). GALNT6 was positively correlated with CCDC88C protein abundance in the normal breast and breast cancer tissues, indicating that GALNT6 might be associated with expression patterns of CCDC88C in breast cancer. Our data demonstrated that GALNT6 maintained CCDC88C stability by promoting its O-linked glycosylation, and the modification was critical for the pro-metastatic potential of CCDC88C. CCDC88C also could mediate the pro-metastatic potential of GALNT6 in breast cancer. Collectively, our findings uncover that CCDC88C may increase the risk of breast cancer metastasis and elucidate the underlying molecular mechanisms.

**Supplementary Information:**

The online version contains supplementary material available at 10.1186/s12935-024-03413-2.

## Introduction

Breast cancer is the most commonly diagnosed malignant tumor in women worldwide [1] and it is generally classified based on classical immunohistochemistry markers such as estrogen receptor (ER), progesterone receptor (PR), and human epidermal growth factor receptor 2 (HER2): luminal (A and B), HER2-positive, and triple-negative breast cancer [2]. Despite advances in surgery, chemotherapy, endocrine therapy, and radiotherapy over the past decade, the incidence and mortality of BC remain high. It was estimated that the number of new patients with breast cancer and new deaths induced by breast cancer reached 2,261,419 and 684,996 in 2020, constituting 11.7% of all cancer cases and 6.9% of all cancer deaths [1]. Unpredictable progression, such as recurrence and metastasis, is the predominant cause of poor prognosis of breast cancer patients and it remains a major clinical challenge. A better understanding of the molecular mechanism underlying breast cancer metastasis is still necessary for the development of effective therapies.

Coiled-coil domain containing 88C (CCDC88C), also known as Dishevelled-associating protein with a high frequency of leucine residues (Daple), is a multimodular signal transducer discovered as a binding partner of the Wnt signaling protein Dishevelled (Dvl) [3]. CCDC88C possesses a Gα-binding and activating (GBA) motif, a Frizzled (FZD)-binding domain, and a PZD-binding motif (PBM) [4]. CCDC88C binds the PDZ domain of Dvl via the PBM, and the interaction activates the non-canonical Wnt/Rac pathway and regulates the formation of lamellipodia and cell motility [3, 5]. CCDC88C antagonizes the canonical Wnt signaling by competing with Dvl for binding to FZD7R to activate Gαi and enhance the non-canonical Wnt signaling [4]. The GBA motif and the FZD-binding domain are involved in the formation of FZD7R-CCDC88C-Gαi3 complexes [4]. In colorectal cancer, CCDC88C is highly expressed in metastasized tumor cells of patients, and its high expression is linked to a poor prognosis for patients with metastatic colorectal cancer, implying that CCDC88C may be involved in tumor metastasis in colorectal cancer [5]. However, the role of CCDC88C in breast cancer metastasis remains unclear.

Glycosylation is a complex and ubiquitous post-translational modification, and the modification exerts various roles in physiological and pathological conditions. Protein glycosylation is mainly divided into multiple forms, including O-linked glycosylation and N-linked glycosylation. O-linked glycosylation often occurs in the Golgi and Endoplasmic Reticulum [6]. O-glycans are generated by adding N-acetylgalactosamine (GalNAc), N-acetylglucosamine, fucose, glucose, xylose, mannose, or galactose to serine (Ser) and threonine (Thr) residues of proteins [7]. O-GalNAc glycosylation (Mucin-type O-glycosylation) is initiated by a family of 20 homologous genes encoding polypeptide GalNAc-transferases (ppGALNTs), and the enzymatic process is initiated in in the Golgi [6, 8]. Abnormal O-glycosylation of proteins is linked to the development of cancers, including tumor growth, tumor metastasis, and immune escape [9–12]. GALNT6 functions as an oncoprotein in breast cancer [11, 13]. Multiple proteins, including mucin 1 [11], estrogen receptor alpha [14], lectin galactoside‑binding soluble 3 binding protein [13], and α2-macroglobulin [15], have been identified as the substrate of GALNT6 in breast cancer. It is necessary to identify other substrates for GALNT6, and to understand the underlying mechanism of GALNT6 in breast cancer metastasis.

In this study, we aimed at testing whether CCDC88C played a vital role in breast cancer metastasis, what was important for CCDC88C to exert its biological functions in breast cancer metastasis, and whether CCDC88C served as a substrate of GALNT6.

## Materials and methods

### Antibodies

Rabbit anti-CCDC88C antibody (Cat#A302-951A) was purchased from ThermoFisher Scientific (USA). Rabbit anti-E-cadherin (Cat#AF0131), rabbit anti-zonula occludens protein 1 (ZO-1) antibody (Cat#AF5145), rabbit anti-phospho c-JUN (p-c-JUN, Ser 63) antibody (Cat#AF3089), rabbit anti-p-c-JUN (Ser 73) antibody (Cat#AF3095), rabbit anti-c-JUN antibody (Cat#AF6090), and rabbit anti-HA antibody (Cat#T0050) were purchased from Affinity (China). Rabbit anti-vimentin antibody (Cat#A19607) and rabbit anti-DDDDK-Ta (Flag) antibody (Cat#AE063) were purchased from Abclonal (China). Mouse anti-GALNT6 antibody (Cat#sc-100755) was purchased from Santa Cruz (USA). Mouse anti-GAPDH antibody (Cat#60004-1-Ig) and rabbit anti-Cell Migration Inducing Hyaluronidase 1 (CEMIP) antibody (Cat#21129-1-AP) were purchased from Proteintech (USA). Secondary antibodies including HRP-conjugated goat anti-rabbit IgG (Cat#SE134) and goat anti-mouse IgG (Cat#SE131) were purchased from Solarbio (China).

### Cell culture

Human breast cancer cell lines BT549 and SKBR3 were purchased from the Cellverse Bioscience Technology Co., Ltd. (China). BT549 and SKBR3 cells were grown in Roswell Park Memorial Institute (RPMI) 1640 medium (Solarbio, China) and McCOY's 5A medium (Servicebio, China) supplemented with 10% fetal calf serum (TIANHANG, China) at 37 °C with 5% CO_2_ in a humidified atmosphere, respectively.

### Plasmids

Sequences of *GALNT6* gene, short hairpin RNAs (shRNAs) targeting *CCDC88C* (sh-CCDC88C), and small interfering RNAs (siRNAs) targeting *GALNT6* (si-GALNT6), *CEMIP* (si-CEMIP), and *JUN* (si-JUN) were synthesized by GENERAL BIOL (China). WT or mutant *GALNT6* was cloned into the pCDNA3.1-HA vector. The sh-CCDC88C sequence was cloned into the pRNA-H1.1/Neo vector. The pCDNA3.1, the pCDNA3.1-eGFP, and the pcDNA3.1-3xFlag vectors expressing WT or mutant *CCDC88C* were purchased from YouBio (China).

### Cell transfection and generation of stable cell lines

BT549 and SKBR3 cells were transfected with plasmids overexpressing CCDC88C or shRNAs targeting CCDC88C using Lipofectamine™ 3000 (Invitrogen, USA). After 24 h, the cells were selected in complete medium containing 500 μg/mL of G418 (BT549: 400 μg/mL; SKBR3: 300 μg/mL) to generate stable overexpression or knockdown of CCDC88C cell lines. BT549 cells with/without stable overexpression of CCDC88C were transfected with siRNAs targeting CEMIP or JUN or plasmids overexpressing GALNT6 using Lipofectamine™ 3000 (Invitrogen, USA). SKBR3 cells were transfected with siRNAs targeting GALNT6 using Lipofectamine™ 3000 (Invitrogen, USA).

### Wound healing assay

A total of 5 × 10^5^ cells/well were seeded and incubated overnight. A 200-μL pipette was used to scratch the cell surface. After 24 h- (BT549 cells) or 12 h- (SKBR3 cells) incubation in serum-free medium, the plates were photographed. The migration rate was calculated using the following formula: (the wound width at 0 h—the wound width at 24/12 h)/the wound width at 0 h.

### Transwell assay

The membrane of transwell chamber was coated with Matrigel (Corning, USA) to form a matrix barrier. Cells (5 × 10^4^ cells/well) in serum-free medium were seeded into the upper chamber, and 700 μL of medium containing 10% fetal calf serum was added into the lower chamber for 24 h. The cells invaded into the lower side of the membranes were stained by 0.5% crystal violet (Amresco, USA).

### Phalloidin staining

Approximately 5 × 10^4^ cells were seeded onto glass coverslips. The cells were fixed with 4% paraformaldehyde for 15 min and permeabilized with 0.1% Triton X-100 (Beyotime, China) for 30 min. The cells were incubated with TRITC-phalloidin (Solarbio, China) and the nuclei were stained with DAPI (Aladdin, China). Antifading mounting medium (Solarbio, China) was used to protect the fluorescent signal.

### Cell counting kit 8 (CCK8) assay

CCK8 assay was performed using the commercial assay kit produced by Solarbio (China). Approximately 5 × 10^3^ cells were seeded into a 96-well culture plate. After 0, 24, 48, and 72 h, 10 μL CCK8 reagent per well was added. The optical value at 450 nm was detected after 2 h-incubation.

### Immunoblotting and vicia villosa (VVA) lectin blotting

Cells were lysed on ice for 5 min with RIPA lysis buffer (Solarbio, China) containing 1 mM PMSF (Solarbio, China). The lysates were centrifuged at 10,000 × *g* for 5 min at 4 °C. The supernatant was kept as the protein extract and its concentration was determined by the BCA assay kit (Solarbio, China). Sodium dodecyl sulfate polyacrylamide gel electrophoresis (SDS-PAGE) was performed using 8% or 10% polyacrylamide gels. Proteins were transferred to a polyvinylidene difluoride (PVDF) membrane (Millipore, China). The membrane was incubated with primary antibodies or biotinylated VVA lectin (Cat#B-1235, Vector Laboratories, USA) overnight at 4 °C and then it was incubated for 1 h at 37 °C with HRP-conjugated secondary antibodies for or HRP-conjugated streptavidin (Cat#SA00001-0, Proteintech, USA) for biotin detection. Finally, the membrane was developed with ECL western blotting substrate (Solarbio, China).

### Quantitative real-time PCR (qRT-PCR)

Total RNA harvested from cultured cell lines using TRIpure reagent (BioTeke, China) was reverse transcribed to cDNA using the BeyoRT II M-MLV reverse transcriptase (Beyotime, China). qRT-PCR was run in 20 µL reactions using SYBR Green (Solarbio, China) with the following reactions conditions: 95 °C for 5 min, followed by 40 cycles of 95 °C for 10 s, 60 °C for 10 s, and 72 °C for 15 s, 72 °C for 90 s, and 40 °C for 60 s. Expression values were normalized to the *GAPDH* gene using the ΔΔCt quantification method. The following primer pairs were used for qRT-PCR analysis: *CCDC88C*-F 5′- AAGGAGACGGAGAACGAAA -3′, *CCDC88C*-R 5′- GCTGTGCTGGCGGATGA -3′; *JUN*-F 5′- ACGACCTTCTATGACGATGC -3′, *JUN*-R 5′- CCGTTGCTGGACTGGATT -3′; CEMIP-F 5′- TGTATGGAAGGGCTGAT -3′, *CEMIP*-R 5′- GATGACTGTGCCTGATTT -3′; *GALNT6*-F 5′- TGCCATCAAGAACCTCG -3′, *GALNT6*-R 5′- CAATTCCCATTCCTCGTC -3′.

### Mouse metastasis models

All experiments involving animals were approved by the Ethics Committee of the Second Affiliated Hospital of Zhejiang University. Animal experiments were conducted according to the *Guide for the Care and Use of Laboratory Animals*. Six-week-old female balb/c nude mice were purchased from Huachuang Sino (China). Mice were maintained in a specific-pathogen-free facility (temperature: 22 ± 1 °C; humidity: 45–55%; light–dark cycle: 12 h/12 h) wit ad libitum access to diet and water.

For experimental lung metastasis, BT549 cells were injected into nude recipients via tail vein (5 × 10^5^ cells per mouse). The mice were euthanized at 35 days after injection and then the lungs were dissected. For experimental liver metastases, BT549 cells were intrasplenically injected into nude recipients (1 × 10^6^ cells per mouse). After 5 min, the spleen was removed to prevent early death due to a high tumor load in spleen. The mice were euthanized 30 days after injection and the livers were dissected. The lung and liver metastasis were monitored by the IVScope8200 small animal imaging system (Clinx, China). The metastatic nodules on the lung surface and the liver surface were counted. The liver and the lung tissues were collected for further evaluation. The tumor tissues were fixed in 10% formalin and embedded in paraffin for hematoxylin and eosin (H&E) staining.

### H&E staining

Paraffin-embedded lung and liver tissues were sectioned (5 µm), deparaffinated, re-hydrated, and stained with hematoxylin (Solarbio, China) and eosin (Sangon, China). The pathological changes were observed under a BX53 microscope (OLYMPUS, Japan).

### mRNA sequencing (mRNA-seq) and identification of differentially expressed genes (DEGs)

mRNA-seq experiments were performed with three biological replicates of BT549 cells with ev and CCDC88C-oe plasmids. Total RNA was extracted using TRIzol following the manufacturer’s instructions. RNA samples were quantified using the Nanodrop (ThermoFisher Scientific, USA) and the 5400 bioanalyzer (Agilent, USA), and the quality was evaluated using the 5400 bioanalyzer. Poly-A-tailed mRNA was isolated from total RNA using Oligo(dT) coupled to magnetic beads. The isolated mRNA was fragmented in fragmentation buffer and then reverse-transcribed with M-MuLV reverse transcriptase. RNA was removed by RNaseH. Next, the double-stranded cDNA was subjected to end-repair, 'A' base addition, and sequencing adapters addition. The AMPure XP Beads were used to select the cDNA fragments of 370–420 bp. PCR amplification was performed for DNA enrichment, and then the cDNA fragments were purified with AMPure XP Beads.

The concentration of the libraries was quantified using the Qubit2.0 fluorometer (ThermoFisher Scientific, USA). The libraries were diluted to 1.5 ng/μL, followed by analysis of insert sizes using the Agilent BioAnalyzer 2100 (Agilent, USA). The libraries were sequenced using the Illumina NovaSeq 6000 (Illumina, USA). The quality of the libraries was first evaluated. The reads were filtered according to the following criteria: (1) reads that aligned to adaptors, (2) reads with unknown bases, and (3) reads with more than 50% of the number of bases with Qphred ≤ 20.

Clean reads were mapped to the reference genome using the HISAT2 (version 2.0.t). The String Tie (version 1.3.3b) and the FeatureCounts (version 1.5.0-p3) were used to assemble transcripts and quantify the gene expression levels, respectively. The mRNA expression levels were estimated by FPKM values. Differential expression analysis of two groups was performed using the DESeq2 (version 1.20.0). The resulting p value was adjusted (Adjust p value, Adi p) using Benjamini and Hochberg's methods. The mRNAs with threshold values of Adj p ≤ 0.001 and |Log_2_ (fold change, FC)|≥ 2 were considered as DEGs between the two groups.

To uncover the key roles of DEGs, Gene Ontology (GO) enrichment analyses were performed using the clusterProfiler (Version 3.8.1). GO terms were classified into cellular components (CC), molecular functions (MF), and biological processes (BP). Only the TOP20 enriched GO terms with an Adj p < 0.05 were displayed.

### Online data acquisition and identification of DEGs

The microarray datasets GSE30480 and GSE195842 were downloaded from the Gene Expression Omnibus (GEO) online database (https://www.ncbi.nlm.nih.gov/geo/). All primary tumor tissue samples (n = 14) and lymph node metastatic tissue samples (n = 6) collected from breast cancer patients in the GSE30480 dataset were included to analyze the expression levels of CCDC88C. The DEGs in the GSE195842 dataset were identified between the si-JUN group and the sinc group (n = 3 per group) using the GEO2R online tool. Overall survival and recurrence free survival of lymph node metastatic (LN^+^) breast cancer patients with higher or lower levels of CCDC88C mRNA was obtained from the Kaplan–Meier plotter database (http://kmplot.com/analysis/index.php?p=background).

### Co-immunoprecipitation (Co-ip) assay

Cell lysates were harvested using the Native lysis buffer (Solarbio, China) supplemented with 1 mM PMSF and centrifuged at 10,000 × g for 5 min at 4 °C. Protein concentration was determined by the BCA assay kit (Solarbio, China). AminoLink plus coupling resin was washed by adding 200 μL of 1 × coupling buffer. Anti-GALNT6 or anti-CCDC88C antibody was immobilized on AminoLink plus coupling resin for 2 h. After washing, the cell lysates were incubated with the resin and the mixture was slowly oscillated for 2 h. After elution, samples were analyzed by immunoblotting or VVA lectin blotting.

### Cycloheximide (CHX) assay

Cells were collected 0, 2, 4, and 6 h after CHX treatment (20 μg/mL) and the stability of CCDC88C was evaluated by immunoblotting.

### Dual luciferase reporter assay

Stable BT-549 cell lines with CCDC88C overexpression were transfected with pAP1-Ta-luc plasmids (Beyotime, China) and pRL-TK plasmids using Lipofectamine™ 3000 (Invitrogen, USA). The activity of firefly luciferase and renilla luciferase was measured by the dual luciferase reporter gene assay kit (KeyGEN, China).

### Statistical analysis

For the statistical analyses, GraphPad software was used. Student’s t-test and one-way or two-way analysis of variance (ANOVA) were used if the data were normally distributed. A minimum of three biological independent samples were required for statistical significance. Data were presented as mean ± standard deviation (SD). Statistical significance is indicated as p < 0.05.

## Results

### CCDC88C is related to breast *cancer* metastasis

**Fig. 1:**
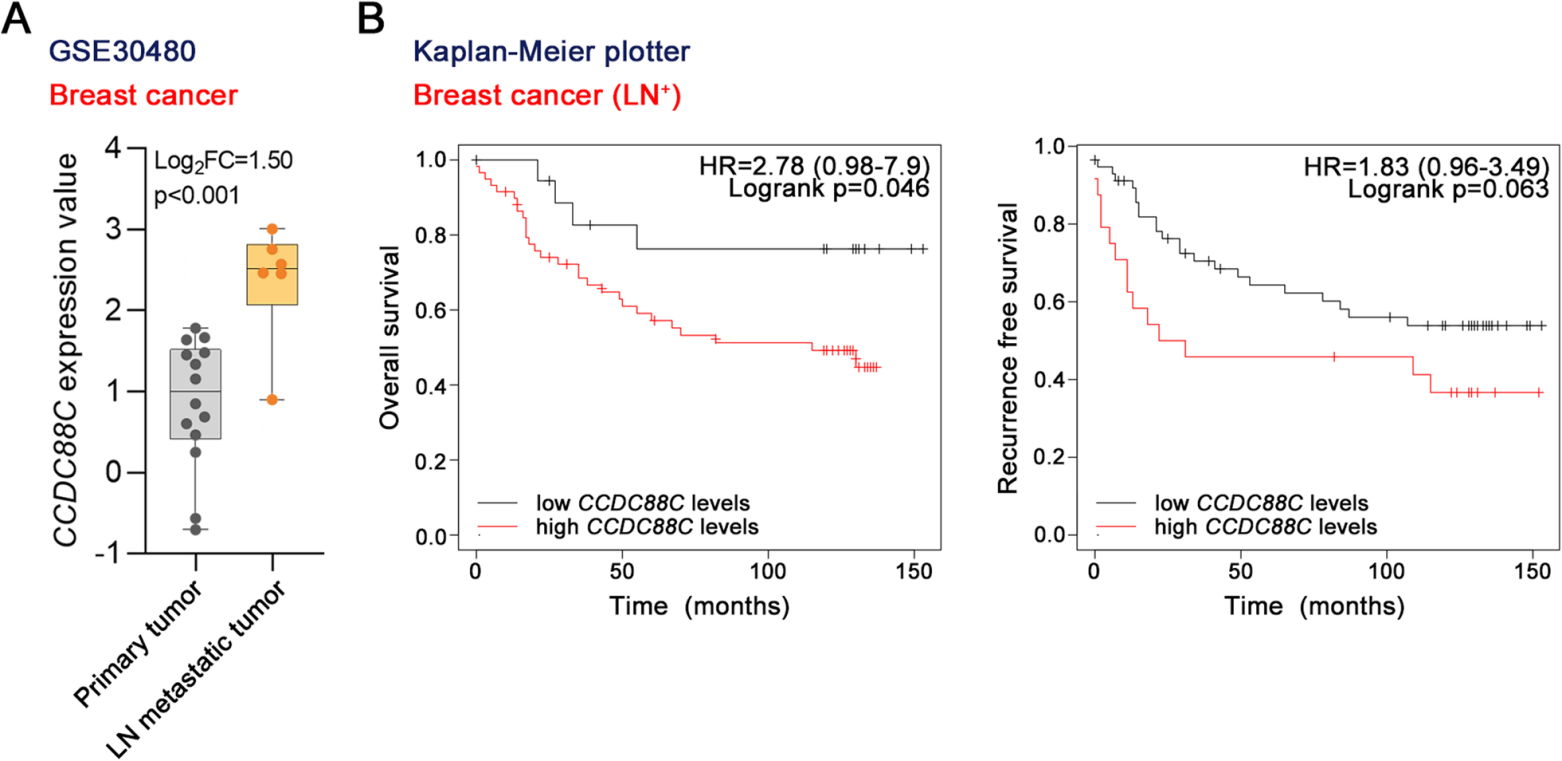
Higher levels of *CCDC88C* mRNA were closely correlated with poor prognosis of LN^+^ breast cancer patients. **A** Levels of *CCDC88C* mRNA in the primary tumor (n = 14) and LN metastatic tumor (n = 6) tissues of breast cancer in the GSE30480 dataset. The upper and lower sides of the box are the third and the first quartiles, and the central line within the box depicts the median. **B** Overall survival and recurrence free survival of LN^+^ breast cancer patients with higher or lower levels of *CCDC88C* mRNA according to the Kaplan–Meier plotter database. *CCDC88C* coiled-coil domain containing 88C. *FC* fold change. *LN*^*+*^ lymph node positive

A microarray dataset GSE30480 revealed that *CCDC88C* expression was higher in lymph node metastatic tissues than in primary tumor tissues from breast cancer patients (Fig. 1A). The overall survival and recurrence free survival were analyzed following the stratification based on *CCDC88C* expression (high and low). As shown in Fig. 1B, the overall survival and recurrence free survival time for lymph node metastatic breast cancer patients with high *CCDC88C* expression were shorter than those for patients with low *CCDC88C* expression. These findings suggest that high *CCDC88C* expression is associated with a poor prognosis for lymph node metastatic breast cancer patients.

### CCDC88C enhances breast *cancer* metastasis in vitro and in vivo

**Fig. 2:**
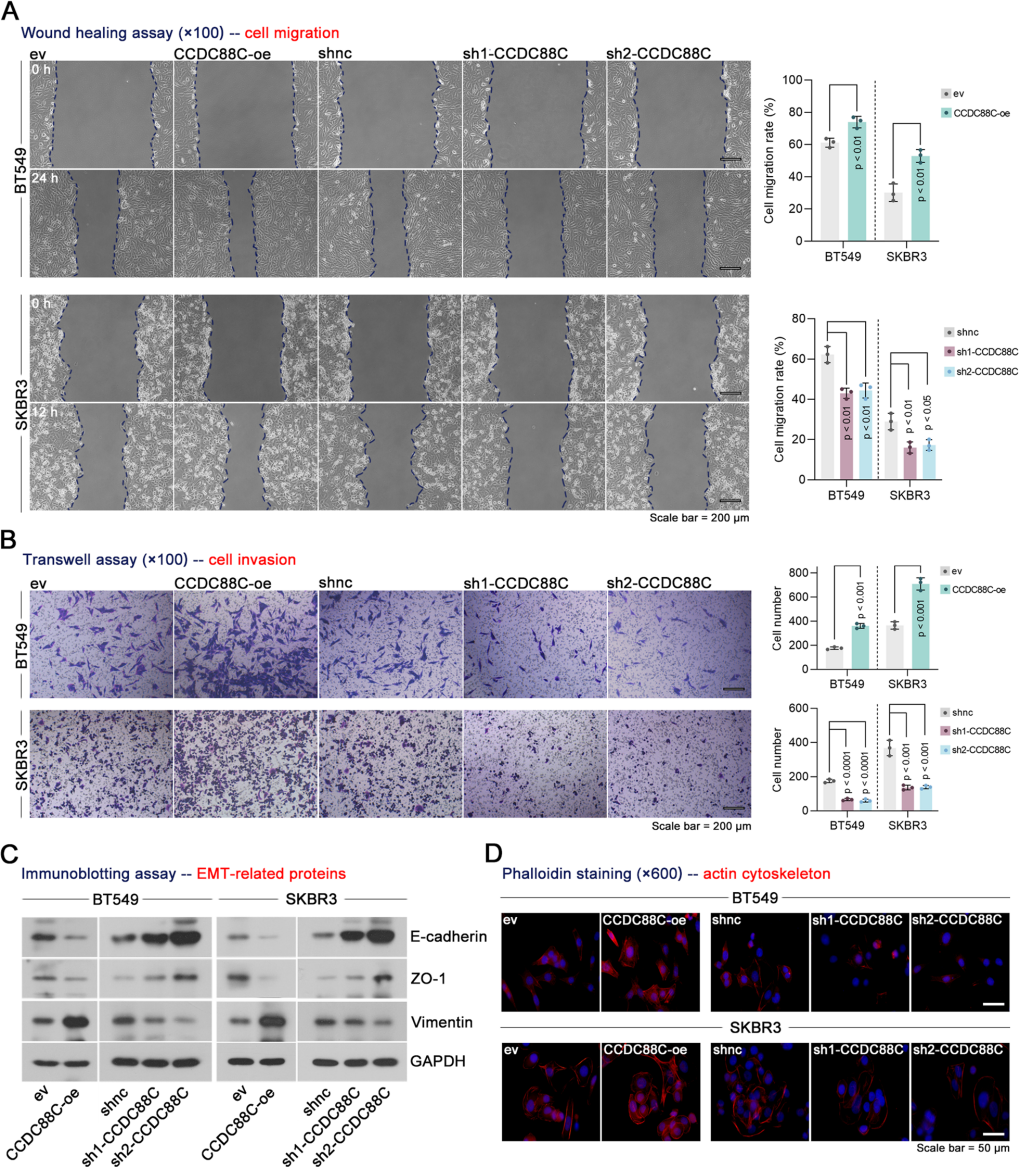
Effect of CCDC88C on cell motility in breast cancer in vitro. **A** Cell migration was measured by wound healing assays (× 100 magnification). Scale bar: 200 μm. **B** Cell invasion was determined by transwell assays (× 100 magnification). Scale bar: 200 μm. **C** Immunoblotting was used to detect EMT-related proteins, including E-cadherin, ZO-1, and Vimentin. **D** Phalloidin staining was performed to detect actin cytoskeleton (× 600 magnification). Scale bar: 50 μm. Data are expressed as the mean ± SD. *CCDC88C* coiled-coil domain containing 88C. *ev* empty vectors. oe, overexpression. *shRNA* short hairpin RNA. *shnc* negative control shRNA. *EMT* epithelial-mesenchymal transition

To investigate the role of CCDC88C in cell motility in vitro, we utilized two independent shRNAs or CCDC88C overexpression plasmids to perform the knockdown or overexpression of *CCDC88C* in the BT549 and SKBR3 cell lines (Supplementary Fig. 1A, B). Stable cell lines with *CCDC88C* knockdown and overexpression were developed (Supplementary Fig. 1C, D). Overexpression of *CCDC88C* promoted the migration and invasive abilities of breast cancer cells (Fig. 2A, B). Epithelial-mesenchymal transition (EMT) was increased by *CCDC88C* overexpression, as evidenced by down-regulation of epithelial markers including E-cadherin and ZO-1 and up-regulation of a mesenchymal marker Vimentin (Fig. 2C). Loss of *CCDC88C* showed an opposite effect on cell migration, cell invasion, and EMT of breast cancer cells (Fig. 2A–C). Actin cytoskeleton underlies key cellular processes, such as cell motility. The phalloidin staining demonstrated that overexpression of *CCDC88C* resulted in increases in actin cytoskeleton in breast cancer cells, whereas depletion of *CCDC88C* had the opposite effect (Fig. 2D). CCK8 assays were used to measure cell viability. It was observed that CCDC88C had no significant effect on cell proliferation (Supplementary Fig. 2).

**Fig. 3:**
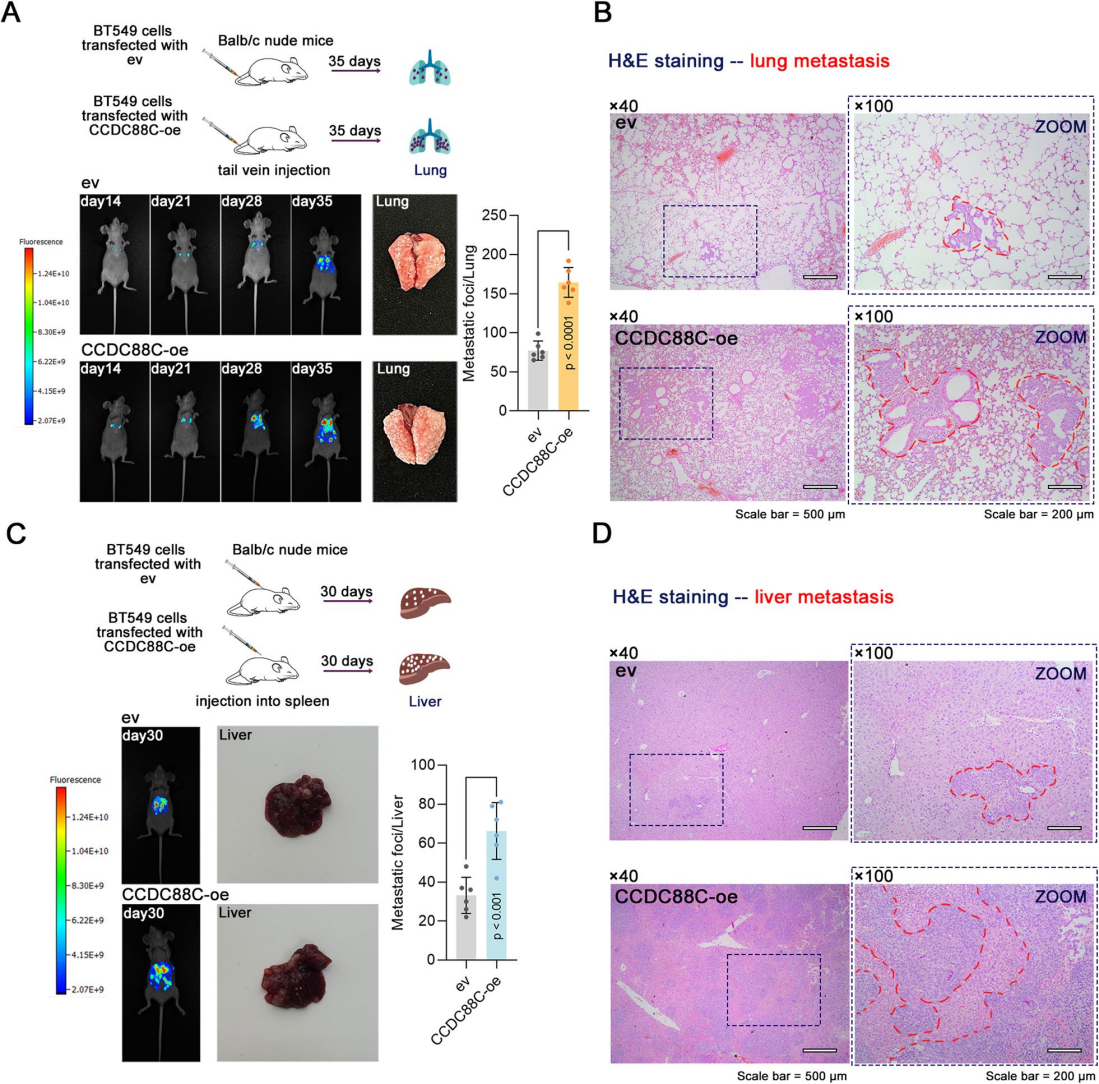
Effect of CCDC88C on lung and liver metastasis in breast cancer in vivo. BT549 cells with stable expression of CCDC88C were injected into nude mice via tail vein. **A** Representative images of fluorescence signals at the indicated time points after cell injection. Lungs were harvested at 35 days after cell injection and the metastatic foci were counted. **B** The lung metastasis was evaluated by H&E staining (× 40 magnification, scale bar: 500 μm; × 100 magnification, scale bar: 200 μm). Red dashed line, metastatic foci. BT549 cells with stable expression of CCDC88C were injected into the spleen of nude mice. The spleen was removed 5 min after injection. **C** Representative images of fluorescence signals at 30 days after cell injection. Livers were harvested at 30 days after cell injection and the metastatic foci were counted. **D** The liver metastasis was evaluated by H&E staining (× 40 magnification, scale bar: 500 μm; × 100 magnification, scale bar: 200 μm). Red dashed line, metastatic foci. Data are expressed as the mean ± SD. *CCDC88C* coiled-coil domain containing 88C. *ev* empty vectors. *oe* overexpression. *H&E* haematoxylin and eosin. Mouse (doi.org/10.5281/zenodo.3925977), Syringe (doi.org/10.5281/zenodo.4912419), lung (doi.org/10.5281/zenodo.3926367), and liver (doi.org/10.5281/zenodo.3926109) images from scidraw.io

Next, we further explored the role of *CCDC88C* in breast cancer metastasis in vivo. We injected *CCDC88C*-overexpressing BT549 cells via tail vein to establish a lung metastasis model or into the spleen to establish a liver metastasis model in Balb/c nude mice. The lungs were collected 35 days after tail-vein injection, and the livers were collected 30 days after spleen injection. Results showed that *CCDC88C* overexpression showed a higher metastasis incidence in the lungs and livers and more lung and liver metastatic foci (Fig. 3A–D).

These findings indicate that CCDC88C has the potential to impede breast cancer metastasis.

### CCDC88C up-regulates CEMIP expression via c-JUN and regulates breast *cancer* cell motility via CEMIP

**Fig. 4:**
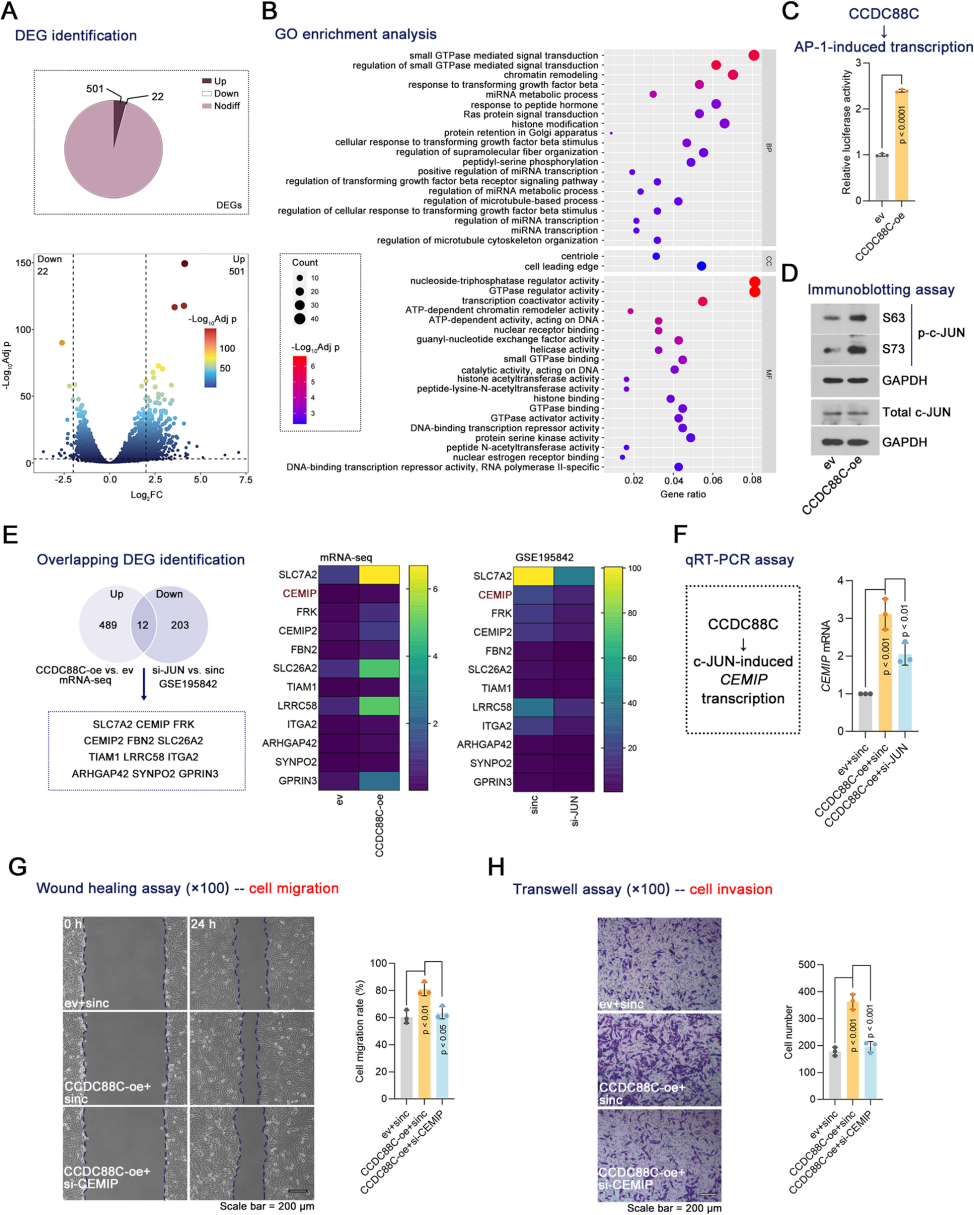
*CEMIP* was identified as a downstream gene of CCDC88C. BT549 cells with stable expression of CCDC88C were used to perform mRNA-seq. **A** The volcano plot showed the DEGs in cells stably transfected with CCDC88C overexpression vectors when compared with cells stably transfected with ev. **B** GO enrichments analysis of the DEGs in cells stably transfected with CCDC88C overexpression vectors. **C** BT-549 cells with stable expression of CCDC88C were transfected with pAP1-Ta-luc and pRL-TK plasmids. After 48 h, the relative luciferase was measured. **D** Immunoblotting was used to detect the level of c-JUN phosphorylation at Ser 63 and Ser 73 and total c-JUN. **E** The Venn diagram showed the overlapping DEGs in breast cancer cells stably transfected with CCDC88C overexpression vectors and breast cancer cells transfected with siRNA targeting *JUN* (siJUN). The heatmap showed 12 overlapping DEG expressions in breast cancer cells stably transfected with CCDC88C overexpression vectors or ev and breast cancer cells transfected with si-JUN or sinc. **F** BT-549 cells with stable expression of CCDC88C were transiently transfected with siJUN. After 48 h, *CEMIP* mRNA was detected using qRT-PCR. (G, H) BT-549 cells with stable expression of CCDC88C were transiently transfected with siCEMIP. After 48 h, Cell migration and invasion were measured by wound healing assays (× 100 magnification) and transwell assays (× 100 magnification), respectively. Scale bar: 200 μm. Data are expressed as the mean ± SD. DEGs, differentially expressed genes. FC, fold change. Adj p, adjust p value. *CCDC88C* coiled-coil domain containing 88C. *ev* empty vectors. *oe* overexpression. *GO* gene ontology. *BP* biological process. *CC* cellular component. *MF* molecular function. *JUN* Jun proto-oncogene, AP-1 transcription factor subunit. *siRNA* small interfering RNA. sinc negative control siRNA. *CEMIP* cell migration-inducing and hyaluronan-binding protein.

To investigate the underlying mechanism triggered by CCDC88C, mRNA-seq was performed on breast cancer cells with or without CCDC88C. DEGs between the two groups were analyzed. We identified 501 up-regulated and 22 down-regulated DEGs (Fig. 4A). GO enrichment analysis showed that DEGs were mainly enriched in the terms related to transforming growth factor beta receptor signaling pathway and small GTPase mediated signal transduction (Fig. 4B). The signaling pathways were associated with cell motility. CCDC88C induces activator protein-1 (AP-1) activation in HEK293 cells [16]. A similar effect was found in breast cancer cell lines (Fig. 4C). It was observed that CCDC88C up-regulated c-JUN phosphorylation but not total c-JUN expression (Fig. 4D). A microarray dataset GSE195842 identified the DEGs in breast cancer cells between the si-JUN and si-nc groups. Twelve overlapping genes were identified between the up-regulated genes in *CCDC88C*-overexpressing cells and the down-regulated genes in *JUN*-knockdown cells compared to the respective control cells (Fig. 4E). *CEMIP* was chosen to verify its involvement in cell motility mediated by CCDC88C. Stable BT549 cell lines with *CCDC88C* overexpression were transfected with the si-JUN that was validated to be effective to knock down *JUN* (Supplementary Fig. 3A). We identified changes in transcriptional levels of *CEMIP* in breast cancer cells induced by CCDC88C that required c-JUN (Fig. 4F). Furthermore, effective depletion of *CEMIP* in CCDC88C-overexpressing cells through transfected with a verified si-CEMIP (Supplementary Fig. 3B, C) resulted in loss of function of CCDC88C in cell migration and invasion (Fig. 4G, H), indicating that the pro-metastatic ability of CCDC88C was CEMIP-dependent. We propose that CCDC88C supports breast cancer metastasis possibly by promoting *CEMIP* transcription.

### GALNT6 maintains CCDC88C stability through regulating its O-GalNAc glycosylation

**Fig. 5:**
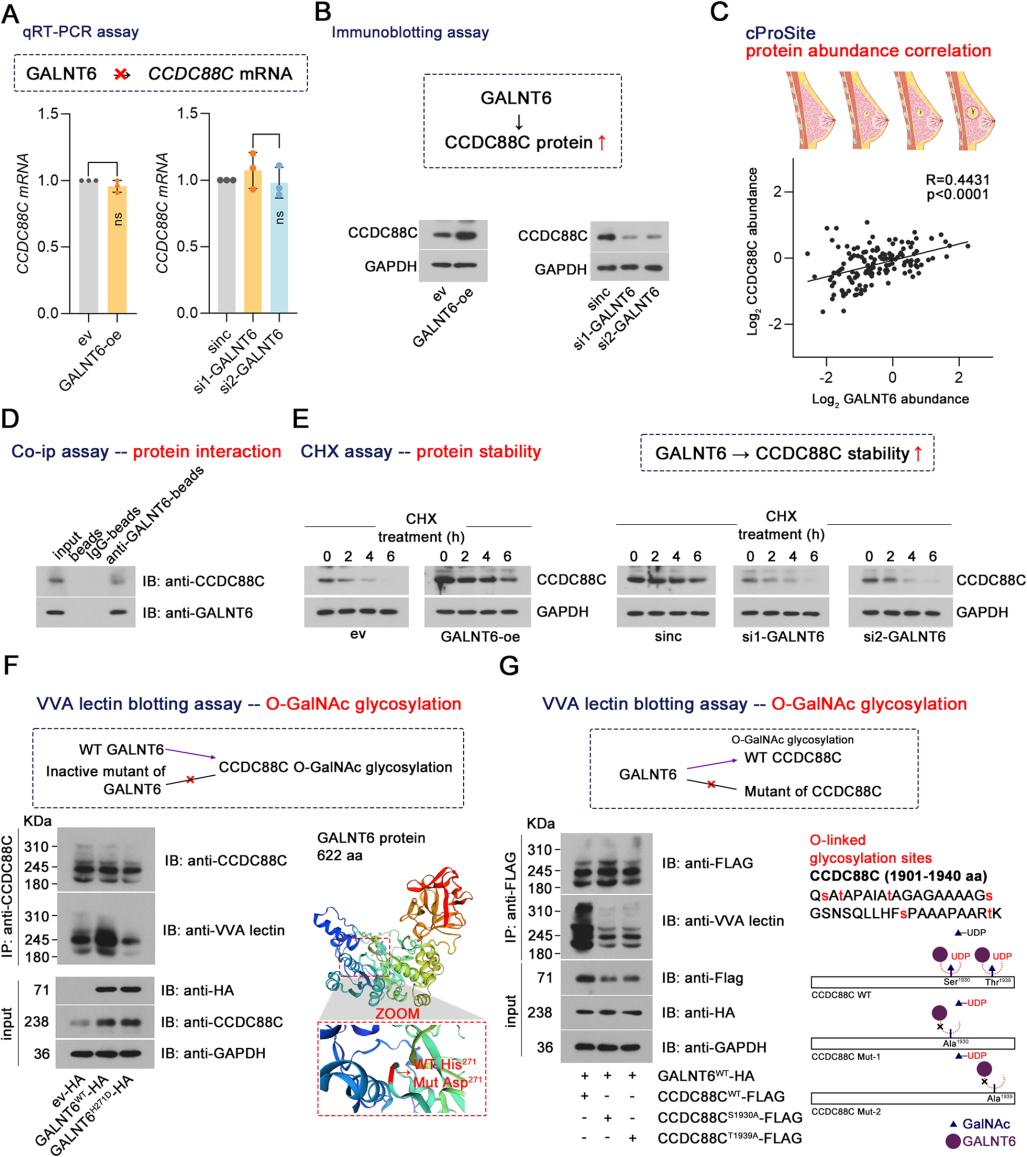
Relationship between GALNT6 and CCDC88C. **A**, **B** BT549 and SKOV3 cells were transiently transfected with GALNT6 overexpression vectors and siRNAs targeting GALNT6 using Lipofectamine™ 3000, respectively. After 48 h, CCDC88C mRNA and protein were detected using qRT-PCR and immunoblotting. **C** The correlation between GALNT6 and CCDC88C protein abundance was analyzed according to the cProSite database. **D** Co-ip was performed to detect the endogenous binding of GALNT6 to CCDC88C. **E** CHX assays were used to detect whether the protein stability of CCDC88C was influenced by GALNT6. **F** BT549 cells were transfected with vectors expressing WT GALNT6 or inactive mutant (H271D) of GALNT6. The GalNAc-type O-glycosylation of CCDC88C was detected by VVA lectin blotting. **G** BT549 cells were co-transfected with vectors expressing WT GALNT6 and vectors expressing WT CCDC88C or mutants (S1930A or T1939A) of CCDC88C. The GalNAc-type O-glycosylation of CCDC88C was detected by VVA lectin blotting. Data are expressed as the mean ± SD. GALNT6, polypeptide N-Acetylgalactosaminyltransferase 6. CCDC88C, coiled-coil domain containing 88C. *ev* empty vectors. oe, overexpression. *siRNA* small interfering RNA. *sinc* negative control siRNA. *CHX* cycloheximide. *VVA* vicia villosa. *WT* wild type. Breast cancer images from bioicons.com. GALNT6 3D structure images from swissmodel.expasy.org

According to the tandem mass spectrometry (MS/MS) analysis published in our previous study [14], CCDC88C was identified as a candidate O-glycosylation substrate of GALNT6. We found that effective knockdown/overexpression of GALNT6 (Supplementary Fig. 3D) had no effect on *CCDC88C* mRNA levels but inhibited/promoted CCDC88C protein levels in breast cancer cell lines (Fig. 5A, B). Analysis of protein abundance correlation based on the cProSite database showed that there was a positive correlation (R = 0.4431, p < 0.0001) between GALNT6 and CCDC88C protein abundance in the normal breast and breast cancer tissues (Fig. 5C). Co-ip assays suggested that GALNT6 could bind to CCDC88C (Fig. 5D). CHX was used to prevent protein synthesis. Upon CHX treatment, CCDC88C protein stability was enhanced by GALNT6 overexpression yet suppressed by GALNT6 knockdown (Fig. 5E). We transfected wild-type GALNT6 or inactive mutant (H271D) of GALNT6 constructs into BT549 cells. As shown in Fig. 5F, WT GALNT6 transformants augmented the O-GalNAc glycosylation of CCDC88C, whereas the inactive mutant had no impact on that of CCDC88C (Fig. 5F). The results implied that the enzyme activity of GALNT6 plays a vital role in the O-GalNAc glycosylation of CCDC88C induced by GALNT6. Previous MS/MS analysis of O-glycosylation substrate of GALNT6 showed that CCDC88C had six O-GalNAc glycosylation sites at Ser1902, Thr1904, Ser1910, Thr1920, Ser1930, and Thr1939. Two sites, including Ser1930 and Thr1939, were selected to validate their involvement in the O-GalNAc glycosylation of CCDC88C induced by GALNT6 (Fig. 5G). We found that a residue substitution at Ser1930 and Thr1939 induced decreases in the O-GalNAc glycosylation and expression of CCDC88C (Fig. 5G and Supplementary Fig. 3E). These findings demonstrate that GALNT6 and O-GalNAc glycosylation of CCDC88C are of importance in maintaining CCDC88C stability in breast cancer cells.

### O-GalNAc glycosylation of CCDC88C is of vital importance for its role in breast cancer cell motility

**Fig. 6:**
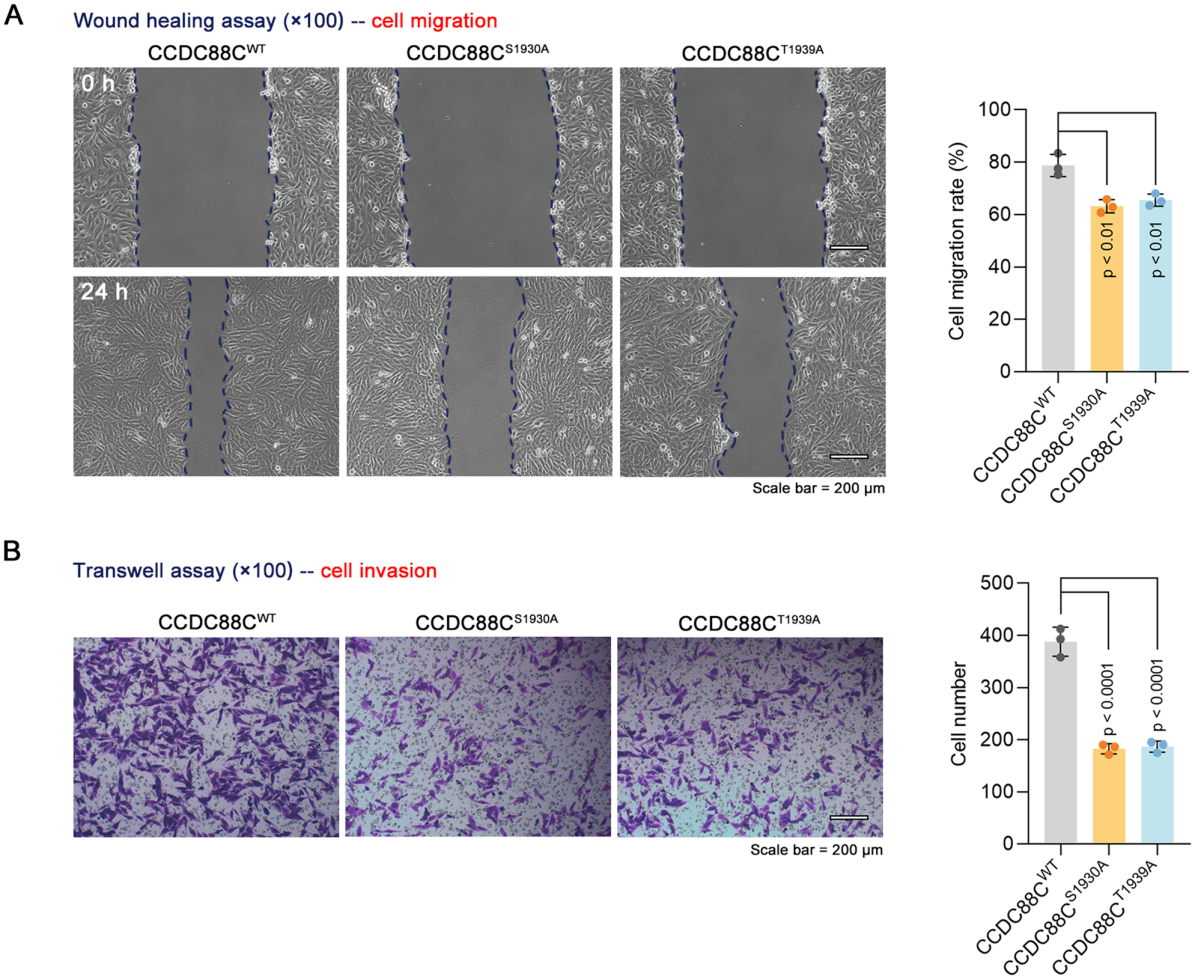
Role of O-glycosylation in the pro-metastatic potential of CCDC88C in breast cancer cells. BT549 cells were transiently transfected with vectors expressing WT CCDC88C or mutants (S1930A or T1939A) of CCDC88C. **A**, **B** After 48 h, Cell migration and invasion were measured by wound healing assays (× 100 magnification) and transwell assays (× 100 magnification), respectively. Scale bar: 200 μm. Data are expressed as the mean ± SD. *CCDC88C* coiled-coil domain containing 88C. *WT* wild type

To investigate the importance of O-GalNAc glycosylation in breast cancer cell motility, we compared the effect of wild-type CCDC88C and mutants (S1930A or T1939A) of CCDC88C on breast cancer cell motility. Mutation of CCDC88C (S1930A or T1939A) impaired the function of CCDC88C (Fig. 6A, B). The findings suggest that the pro-metastatic potential of CCDC88C in breast cancer cells is regulated by O-GalNAc glycosylation.

### CCDC88C is required for breast cancer cell motility mediated by GALNT6

**Fig. 7:**
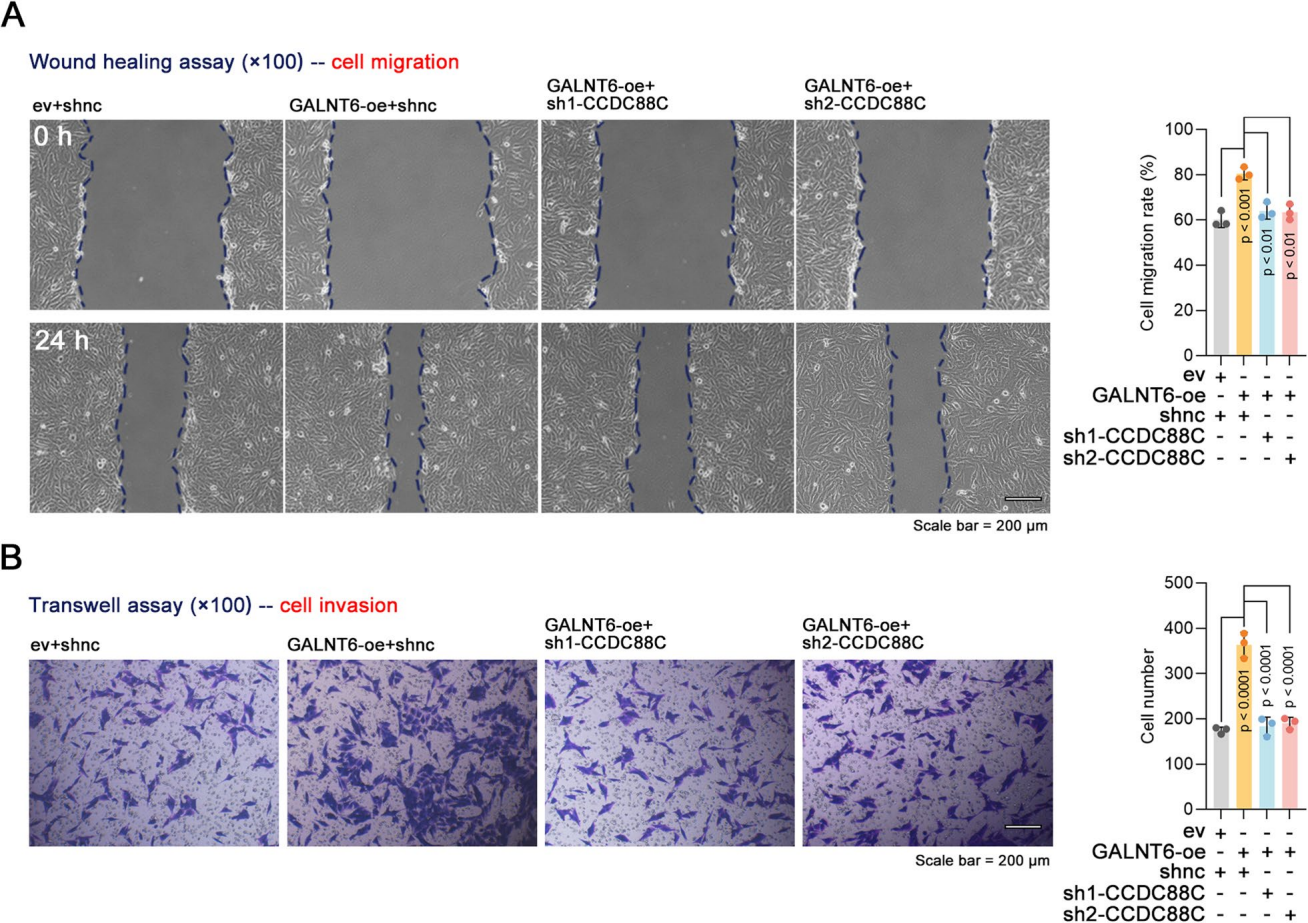
Function of CCDC88C in the enhanced migration and invasion of breast cancer cells induced by GALNT6. BT549 cells with stable knockdown of CCDC88C were transiently transfected with GALNT6 overexpression vectors. **A**, **B** After 48 h, Cell migration and invasion were measured by wound healing assays (× 100 magnification) and transwell assays (× 100 magnification), respectively. Data are expressed as the mean ± SD. *GALNT6* polypeptide N-Acetylgalactosaminyltransferase 6. *CCDC88C* coiled-coil domain containing 88C. *ev* empty vectors. *oe* overexpression. *shRNA* short hairpin RNA. *shnc* negative control shRNA

To determine the role of CCDC88C in the biological function of GALNT6 in breast cancer, GALNT6 was overexpressed in the stable BT549 cell line with CCDC88C knockdown (Supplementary Fig. 3F). We observed that GALNT6 boosted the migration and invasive ability of breast cancer cells (Fig. 7A, B). However, the effects were impaired by CCDC88C knockdown (Fig. 7A, B). It is indicated that GALNT6 supported breast cancer cell motility through CCDC88C.

## Discussion

Breast cancer patients are often diagnosed with metastasis, which results in a poor clinical prognosis. In this study, we confirmed that the mice inoculated with CCDC88C-overexpressed breast cancer cells were more susceptible to lung and liver metastasis. The biological function of CCDC88C in breast cancer metastasis was at least partially mediated by c-JUN-induced *CEMIP* transcription. GALNT6 initiated the O-glycosylation of CCDC88C and thereby promoted CCDC88C protein stability. Moreover, CCDC88C is of importance for GALNT6 to promote breast cancer cell motility.

We identified that CCDC88C was dysregulated during breast cancer metastasis and its high expression was related to a poor prognosis of lymph node metastatic breast cancer patients. Abnormal expression of CCDC88C is also found in colorectal cancer progression, and CCDC88C triggers gastric cancer, colorectal cancer, and cervical cancer cell migration [4, 17]. This is consistent with our results. It is speculated that aberrant expression of CCDC88C may play an important role in metastatic cancer and enhance tumor cells to spread around the body. However, CCDC88C also acts as a tumor suppressor, which inhibits colorectal cancer and cervical cancer cell proliferation [4, 18]. In order to fully understand the mechanism of CCDC88C in breast cancer progression it is necessary to explore whether CCDC88C can suppress breast cancer cell proliferation, which will be investigated in our future work.

Based on mRNA-seq, we identified numerous genes regulated by CCDC88C. CCDC88C is known to trigger activation of Rac that is a member of the Rho family of small GTPases [5]. Consistently, the DEGs induced by CCDC88C overexpression in breast cancer cells mainly enriched in GO terms associated with small GTPase-mediated signal transduction, Ras protein signal transduction, and small GTPase-binding. CCDC88C modulates the Rac-induced reorganization of the actin cytoskeleton to form lamellipodia [5]. In our study, the genes regulated by CCDC88C in breast cancer were also enriched in regulation of microtubule cytoskeleton organization, highlighting the importance of CCDC88C in regulation of actin cytoskeleton. Involvement of transforming growth factor beta in EMT, cell migration, and cell invasion has been widely reported [19]. Many genes enriched in transforming growth factor beta signaling were regulated by CCDC88C. The role of CCDC88C in transforming growth factor beta signal transduction requires further study in our subsequent work.

We provide mechanistic details regarding how CCDC88C is implicated in breast cancer metastasis. c-JUN, encoded by *JUN* gene, is the most extensively studied protein of the AP-1 transcription factor complex. c-Jun N-terminal kinase (JNK) induces c-JUN phosphorylation. CCDC88C activates JNK in gastric cancer cells [17]. We observed that CCDC88C triggered AP-1-induced transcription activation and promoted c-JUN phosphorylation in breast cancer cells. Subsequently, we selected the DEGs regulated by both CCDC88C and c-JUN, and twelve candidate genes were identified. Among these genes, six genes are potential regulator of tumor metastasis, including *CEMIP* [20], *FBN2* [21], *TIAM1* [22], *ITGA2* [23], *ARHGAP42* [24], and *GPRIN3* [25]. *CEMIP*, located on chromosome 15q25, serves as a downstream gene of c-JUN, and its transcription is reduced by knockdown of c-JUN in breast cancer cells [26]. *CEMIP* is upregulated in invasive breast cancer specimens and contributes to a poor prognosis for patients [20]. Mechanistically, *CEMIP* is localized in the endoplasmic reticulum and interacts with the chaperone binding immunoglobulin protein to form a complex [20]. The interaction mediates ER Ca^2+^ leakage and increases in cytosolic Ca^2+^, thereby activating protein kinase C alpha and enhancing breast cancer cell migration [20]. The present study uncovered that the up-regulation of *CEMIP* mRNA induced by CCDC88C required c-JUN, and CCDC88C at least partially depended on CEMIP to drive cell motility.

The Tn antigen (α-GalNAc-O-Ser/Thr) is one of the O-glycans, which is identified by VVA lectin. In breast cancer, previous studies have indicated that higher VVA-positive signals were observed in metastatic cancerous tissues than in primary cancerous tissues and were positively linked to tumor stage, lymphatic invasion, and lymph node metastasis [27, 28]. Also, Tn-positive breast cancer cells exerts higher migration and invasive capacities in vitro and metastatic potentials in vivo than Tn-negative cells [29]. GALNT6 plays a crucial role in the biosynthesis of Tn antigen. The present study suggested that GALNT6 placed GalNAc on CCDC88C and contributed to the maintenance of CCDC88C. The catalytic domain of ppGALNTs is composed of a N-terminal GT1 motif and a C-terminal Gal/GalNAc-T sequence motif [30]. The ppGALNTs contain an aspartate-any residue-histidine (DXH) sequence in the GT1 motif. The conversion of DXH into DXD leads to inactivation of ppGALNTs [30]. In our study, the H211D mutant of GALNT6 had no effect on O-GalNAc glycosylation. The findings further highlighted the importance of GT1 motif in GALNT6-mediated O-GalNAc glycosylation Six O-GalNAc glycosylation sites were identified on CCDC88C (Ser1902, Thr1904, Ser1910, Thr1920, Ser1930, and Thr1939). In the present study, we only confirmed that Ser1930 and Thr1939 are two candidate sites for GALNT6‑dependent O-GalNAc glycosylation sites of CCDC88C. However, the effect of the other four sites on GALNT6‑dependent O-GalNAc glycosylation sites of CCDC88C remains unknown, which needs to be explored in our subsequent work. Likewise, it is also necessary to investigate which sites are linked to the maintenance of the protein stability and biological functions of CCDC88C. These are the limitations for the present study.

## Conclusions

In summary, our data emphasize the essential role of CCDC88C in breast cancer metastasis. Our data also suggest that the function of CCDC88C in breast cancer metastasis requires CEMIP. Furthermore, we confirm that GALNT6 O-glycosylates and stabilizes CCDC88C. Our study uncovers a novel mechanism underlying breast cancer metastasis.

### Supplementary Information

Supplementary Material 1. Fig. S1. Efficiency of CCDC88C overexpression vectors and shRNAs targeting CCDC88C in breast cancer cell lines. (A, B) BT549 and SKOV3 cells were transiently transfected with CCDC88C overexpression vectors or shRNAs targeting *CCDC88C* (5 shRNA, including 2 shRNAs targeting *CCDC88C* CDS and 3 shRNAs targeting *CCDC88C* 3′UTR) using Lipofectamine™ 3000. After 48 h, *CCDC88C* mRNA was detected using qRT-PCR. The shRNAs targeting *CCDC88C* CDS 2 and *CCDC88C* 3′UTR 2 were used for the subsequent experiments. (C, D) BT549 and SKOV3 cells with stable expression or knockdown of CCDC88C were developed. CCDC88C mRNA and protein were detected using qRT-PCR and immunoblotting. CCDC88C, coiled-coil domain containing 88C. ev, empty vectors. oe, overexpression. CDS, coding sequence. 3′UTR, 3′ untranslated region. shRNA, short hairpin RNA. shnc, negative control shRNA.

Supplementary Material 2. Fig. S2. Effect of CCDC88C on cell proliferation in breast cancer in vitro. (A, B) Cell proliferation was measured by CCK8 assays. Data are expressed as the mean ± SD. CCDC88C, coiled-coil domain containing 88C. ev, empty vectors. oe, overexpression. shRNA, short hairpin RNA. shnc, negative control shRNA. ns, no significance.

Supplementary Material 3. Fig. S3. Efficiency of siRNAs targeting *JUN*, *CEMIP*, or *GALNT6*, shRNAs targeting *CCDC88C,* and GALNT6 and CCDC88C overexpression vectors in breast cancer cell lines. (A) BT-549 cells were transfected with siJUN. After 48 h, *JUN* mRNA and c-JUN protein were detected using qRT-PCR and immunoblotting. (B) BT-549 cells were transfected with siCEMIP. After 48 h, CEMIP mRNA and protein were detected using qRT-PCR and immunoblotting. (C) BT-549 cells with stable expression of CCDC88C were transiently transfected with siCEMIP. After 48 h, CEMIP was detected using immunoblotting. (D) BT549 and SKOV3 cells were transiently transfected with GALNT6 overexpression vectors and siRNAs targeting GALNT6 using Lipofectamine™ 3000, respectively. After 48 h, GALNT6 mRNA and protein were detected using qRT-PCR and immunoblotting. (E) BT549 cells were transiently transfected with vectors expressing WT CCDC88C or mutants (S1930A or T1939A) of CCDC88C. After 48 h, CCDC88C protein was detected using immunoblotting. (F) BT549 cells with stable knockdown of CCDC88C were transiently transfected with GALNT6 overexpression vectors. After 48 h, CCDC88C protein was detected using immunoblotting. GALNT6, polypeptide N-Acetylgalactosaminyltransferase 6. CCDC88C, coiled-coil domain containing 88C. ev, empty vectors. oe, overexpression. siRNA, small interfering RNA. sinc, negative control siRNA. shRNA, short hairpin RNA. shnc, negative control shRNA. WT, wild type.

## Data Availability

The data used to support the findings in the present study are available upon reasonable request.
