# Supplementary figures and images for: CCDC88C, an O-GalNAc glycosylation substrate of GALNT6, drives breast cancer metastasis by promoting c-JUN-mediated CEMIP transcription
Source: Cancer Cell Int. 2024 Jul 6;24:237. doi: 10.1186/s12935-024-03413-2 (PMC11227718; doi:10.1186/s12935-024-03413-2)

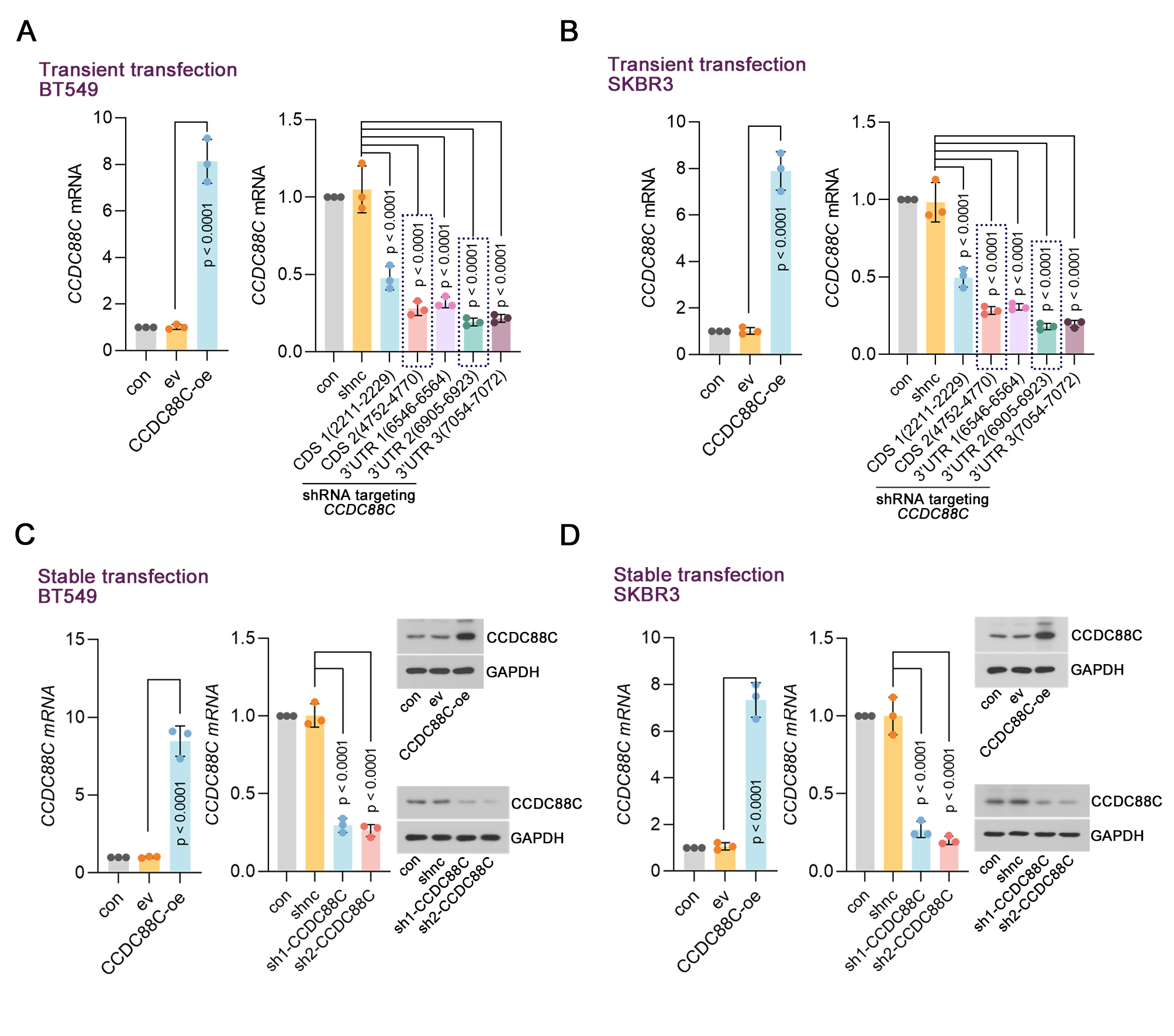

Supplement: Supplementary file 1 — Supplementary Material 1. Fig. S1. Efficiency of CCDC88C overexpression vectors and shRNAs targeting CCDC88C in breast cancer cell lines. (A, B) BT549 and SKOV3 cells were transiently transfected with CCDC88C overexpression vectors or shRNAs targeting CCDC88C (5 shRNA, including 2 shRNAs targeting CCDC88C CDS and 3 shRNAs targeting CCDC88C 3′UTR) using Lipofectamine™ 3000. After 48 h, CCDC88C mRNA was detected using qRT-PCR. The shRNAs targeting CCDC88C CDS 2 and CCDC88C 3′UTR 2 were used for the subsequent experiments. (C, D) BT549 and SKOV3 cells with stable expression or knockdown of CCDC88C were developed. CCDC88C mRNA and protein were detected using qRT-PCR and immunoblotting. CCDC88C, coiled-coil domain containing 88C. ev, empty vectors. oe, overexpression. CDS, coding sequence. 3′UTR, 3′ untranslated region. shRNA, short hairpin RNA. shnc, negative control shRNA. [file 12935_2024_3413_MOESM1_ESM.tif]

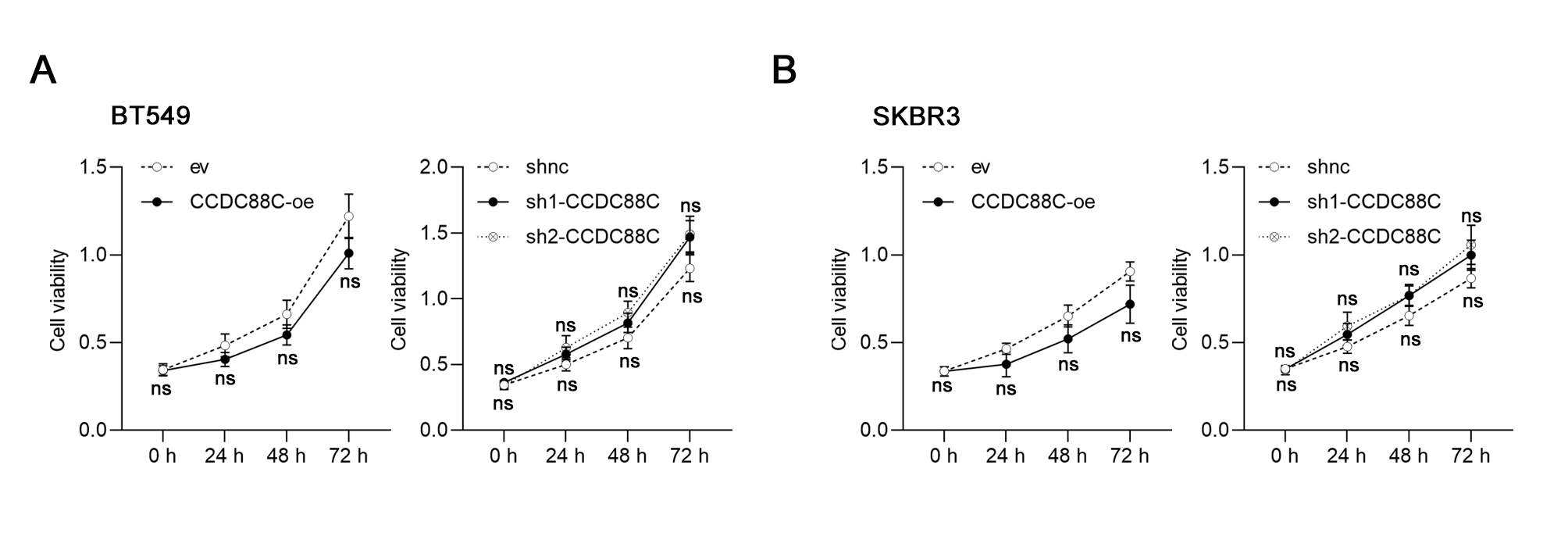

Supplement: Supplementary file 2 — Supplementary Material 2. Fig. S2. Effect of CCDC88C on cell proliferation in breast cancer in vitro. (A, B) Cell proliferation was measured by CCK8 assays. Data are expressed as the mean ± SD. CCDC88C, coiled-coil domain containing 88C. ev, empty vectors. oe, overexpression. shRNA, short hairpin RNA. shnc, negative control shRNA. ns, no significance. [file 12935_2024_3413_MOESM2_ESM.tif]

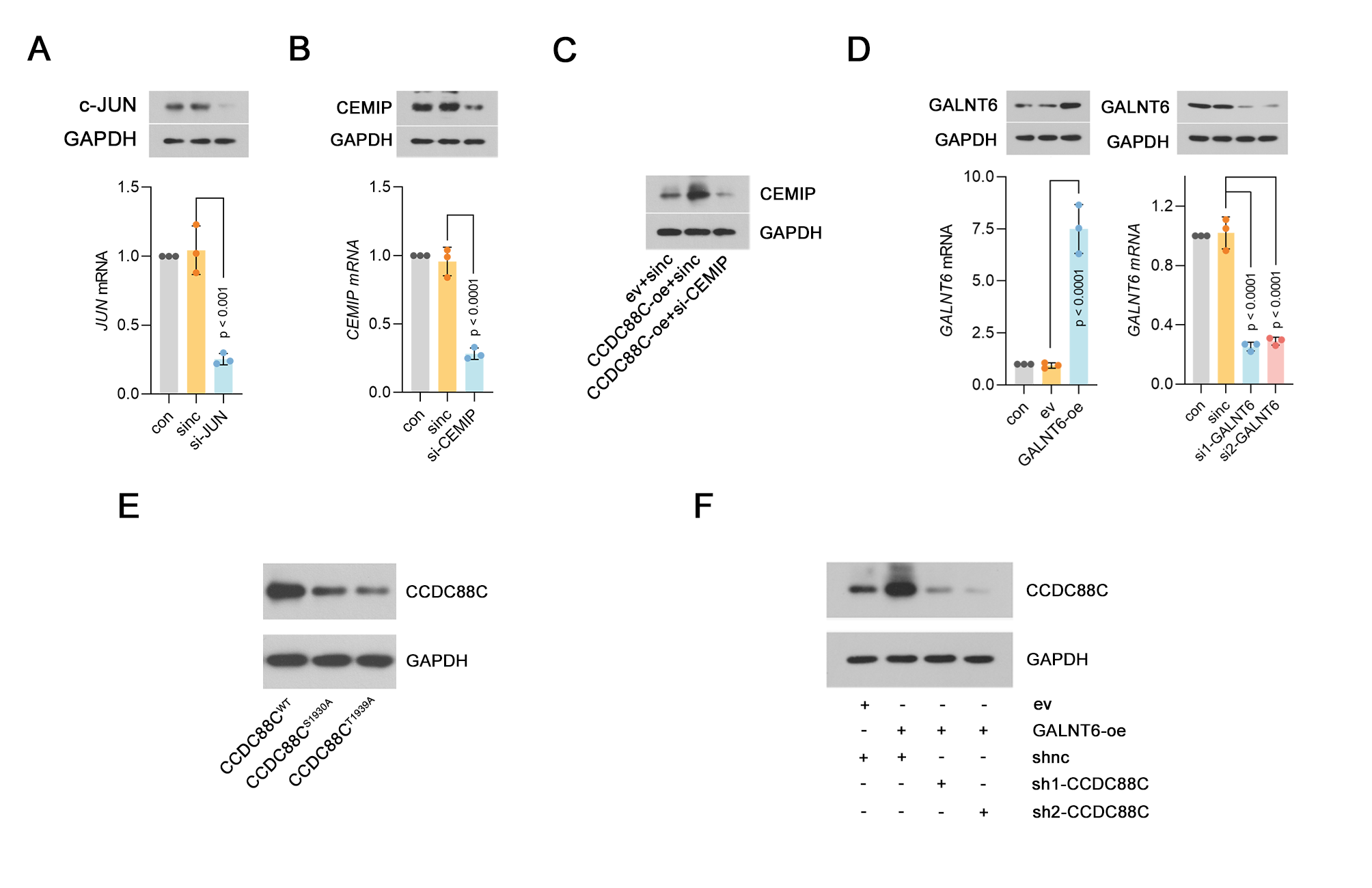

Supplement: Supplementary file 3 — Supplementary Material 3. Fig. S3. Efficiency of siRNAs targeting JUN, CEMIP, or GALNT6, shRNAs targeting CCDC88C, and GALNT6 and CCDC88C overexpression vectors in breast cancer cell lines. (A) BT-549 cells were transfected with siJUN. After 48 h, JUN mRNA and c-JUN protein were detected using qRT-PCR and immunoblotting. (B) BT-549 cells were transfected with siCEMIP. After 48 h, CEMIP mRNA and protein were detected using qRT-PCR and immunoblotting. (C) BT-549 cells with stable expression of CCDC88C were transiently transfected with siCEMIP. After 48 h, CEMIP was detected using immunoblotting. (D) BT549 and SKOV3 cells were transiently transfected with GALNT6 overexpression vectors and siRNAs targeting GALNT6 using Lipofectamine™ 3000, respectively. After 48 h, GALNT6 mRNA and protein were detected using qRT-PCR and immunoblotting. (E) BT549 cells were transiently transfected with vectors expressing WT CCDC88C or mutants (S1930A or T1939A) of CCDC88C. After 48 h, CCDC88C protein was detected using immunoblotting. (F) BT549 cells with stable knockdown of CCDC88C were transiently transfected with GALNT6 overexpression vectors. After 48 h, CCDC88C protein was detected using immunoblotting. GALNT6, polypeptide N-Acetylgalactosaminyltransferase 6. CCDC88C, coiled-coil domain containing 88C. ev, empty vectors. oe, overexpression. siRNA, small interfering RNA. sinc, negative control siRNA. shRNA, short hairpin RNA. shnc, negative control shRNA. WT, wild type. [file 12935_2024_3413_MOESM3_ESM.tif]
